# Being present: oncologists' role in promoting advanced cancer patients' illness understanding

**DOI:** 10.1002/cam4.1389

**Published:** 2018-02-26

**Authors:** Simon M. Cohen, Renee C. Maciejewski, Manish A. Shah, Kelly M. Trevino, Megan J. Shen, Paul K. Maciejewski, Holly G. Prigerson

**Affiliations:** ^1^ Department of Medicine Center for Research on End‐of‐Life Care Weill Cornell Medicine New York City New York; ^2^ Division of Geriatric and Palliative Medicine Department of Medicine Weill Cornell Medicine New York City New York; ^3^ Division of Hematology and Medical Oncology Department of Medicine Weill Cornell Medicine New York City New York; ^4^ Department of Radiology Weill Cornell Medicine New York City New York

**Keywords:** Cancer, end of life, illness understanding, late stage, oncologist, prognosis

## Abstract

Realistic illness understanding is essential to an advanced cancer patient's ability to make informed medical decisions at the end of life. This study sought to determine whether advanced cancer patients better understood the late stage of their cancer if an oncologist, compared to other members of the care team, was present to discuss their scan results. Data were derived from a multi‐institutional, longitudinal cohort study of patients recruited between 2010 and 2015. Patients (*n* = 209) with late‐stage cancers (metastatic cancers that progressed after at least one chemotherapy regimen) were interviewed before and after clinic visits in which scan results were discussed. Patients reported pre‐ and postvisit if their cancer was at a late stage. Postvisit, patients reported if they discussed scan results with an oncologist or another oncology provider (i.e., oncology fellow, oncology resident, nurse practitioner, nurse, physician's assistant, or other). Logistic regression analysis was used to determine if the presence of an oncologist during scan results discussions differentially predicted the patients' likelihood of postvisit late‐stage illness understanding (LSIU). Propensity weighting was used to correct for sociodemographic imbalances between groups, and previsit LSIU and the presence of multiple providers were controlled for in the logistic regression analyses. After propensity‐weighted adjustment and controlling for previsit LSIU and the presence of multiple providers, patients were 2.6 times more likely (AOR = 2.6; 95% CI = 1.2, 6.0; *P* = 0.021) to report that their disease was late stage if an oncologist was present for the scan results discussion compared to if an oncologist was absent. The presence of an oncologist during scan results discussions was associated with a higher likelihood of patients acknowledging being in a late stage of their disease. These results suggest that oncologist involvement in scan results discussions is associated with advanced cancer patients having better prognostic understanding.

## Introduction

For patients to engage in informed end‐of‐life (EoL) decision‐making, they must have a realistic understanding of their prognosis 1, 2, 3. Research has shown that cancer patients who overestimate their life expectancy are more likely to opt for chemotherapy because they have an unrealistic expectation that it may cure them 2, 3. We have found that patients who are hospice eligible, but who do not acknowledge being terminally ill, have lower rates of hospice enrollment 1. Illness understanding in the context of advanced cancer has been linked to preferences for care with comfort as the paramount goal rather than life‐extending therapy 3, 4 and lower rates of chemotherapy use 5. Further, cancer patients who recognize being terminally ill are more likely to receive EoL care consistent with their preferences 4. Nevertheless, recent studies indicate that only 37.7% of patients a median of 4.4 months from death acknowledged that they were terminally ill 6 and only 5% reported completely accurate understanding of their prognosis (i.e., correct response to four measures of illness understanding) 7. These findings highlight the need to improve illness understanding among advanced cancer patients.

Prior studies suggest that *what* is said during discussions with advanced cancer patients about the status of their disease strongly influences whether patients understand their illness 7, 8. However, little is known about the importance of *whom* on the care team delivers this information to patients. Oncologists may be perceived by their patients to have the most advanced medical training (e.g., relative to oncology fellows and nurse practitioners) and to be the highest authority on the care team. The strong influence of authority on behavior is a well‐established psychological phenomenon 9, 10, 11. Thus, it may be expected that oncologists have a stronger influence on cancer patients' illness understanding than other members of the oncology care team when engaging in discussions about disease status.

This study examines whether advanced cancer patients better understand the severity of their illness if a medical oncologist (hereafter oncologist) is present when scan results are discussed in clinic. We hypothesized that discussing scan results with an oncologist would be associated with greater late stage of disease acknowledgment.

## Materials and Methods

### Study sample

The patient sample analyzed in the present report was drawn from the Coping with Cancer II (CwC‐II) study, a National Cancer Institute‐funded, prospective, multi‐institutional cohort study of advanced cancer patients, their caregivers, and their oncology providers, designed to evaluate the relationship between EoL communication and EoL outcomes. Participants were recruited from one of eight medical centers across the United States, which were grouped into three geographical categories: (1) the Mid‐Atlantic/South which included the Meyer Cancer Center at Weill Cornell Medicine (New York, NY), Memorial Sloan Kettering Cancer Center (New York, NY), and Virginia Commonwealth University Massey Cancer Center (Richmond, VA); (2) the Southwest/West which included Parkland Hospital (Dallas, TX), University of New Mexico Cancer Center (Albuquerque, NM), and Pomona Valley Hospital Medical Center (Pomona, CA); and (3) New England which included Dana‐Farber/Harvard Cancer Center (DF/HCC: Dana‐Farber Cancer Institute, Brigham and Women's Hospital, and Massachusetts General Hospital; Boston, MA) and Yale Cancer Center (New Haven, CT).

Criteria for patient eligibility included the following cancers: metastatic pancreaticobiliary, esophagogastric, hepatocellular, lung, or ovarian cancers that progressed on at least one chemotherapy regimen, or metastatic colorectal cancer that progressed on at least two chemotherapy regimens. Eligibility criteria also included age ≥21 years and the ability to complete study interviews. Patients who were cognitively impaired (e.g., rater‐perceived inability to provide reliable and valid responses to survey questions) or had received palliative care prior to enrollment were excluded. All study participants provided written informed consent. The institutional review boards of all participating institutions approved the study's conduct.

**Figure 1:**
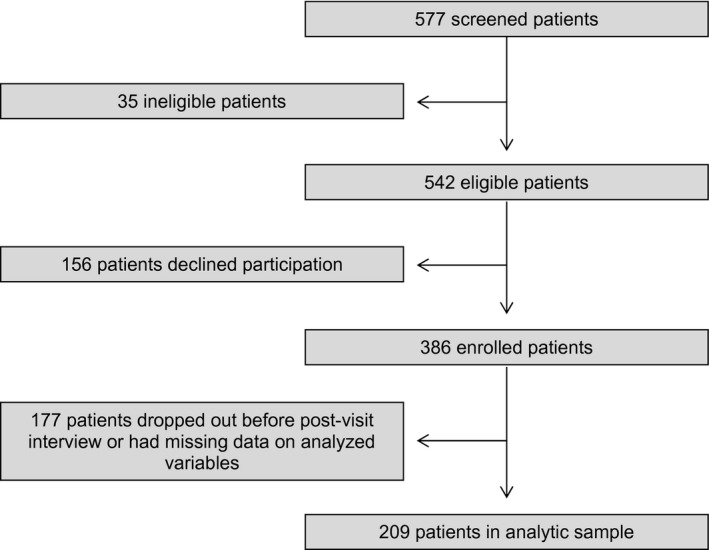
Flow diagram of patients.

Interviews were conducted between February 2010 and February 2015. Patients were interviewed at study entry (previsit) and then again within a week after an oncology visit in which recent scan results to evaluate potential disease progression were discussed (postvisit). These were not the first scan results that indicated patients had advanced cancer, as patients had to have previous scan results that showed progression on at least one chemotherapy regimen in order to be eligible for the study. The interviews were conducted by research assistants (RAs) who underwent extensive training on how to conduct the interviews in a sensitive and brief manner. The training was administered by an experienced, trained RA who practiced these techniques, and RAs were only permitted to interview subjects after demonstrating reliability, accuracy, and sensitivity. A total of 386 patients enrolled in the study (Fig. 1), and the present report analyzes data from the 209 CwC‐II participants who completed these pre‐ and postvisit interviews and had complete data on study measures. A total of 177 participants were not included in the study sample because either they dropped out of study after the previsit interview or there were missing data on study variables collected at the pre‐ or postvisit interview. In dying patient cohorts such as the one in this study, the severity of illness and proximity to death of patients contribute to a high dropout rate 6. Relative to the analytic sample, the participants not included in study analyses had a lower level of education and were more likely to be non‐white, Latino, and from the Southwest/West (Table S1). Importantly, there were no differences between the two groups in the presence of an oncologist during scan discussions, previsit late‐stage illness understanding (LSIU) or postvisit LSIU—the primary outcome measure.

### Measures

#### Patient sociodemographic characteristics

Patients provided information on their age, education, gender, race, ethnicity, health insurance status, and marital status. Patients' clinic site and primary cancer were also documented.

#### Late‐stage illness understanding

Patients' late‐stage illness understanding (LSIU) was assessed in the interviews at pre‐ and postvisit by asking: “What stage is your cancer?” Response options were: (1) no evidence of cancer, (2) early stage of cancer, (3) middle stage of cancer, (4) late stage of cancer, (5) end stage of cancer, and (6) don't know. Acknowledgment of late stage of cancer was coded “1” for response options 4 and 5, and “0” for response options 1, 2, 3, and 6. LSIU was shown to be a valid assessment of prognostic understanding and significantly and closely associated with other measures of this construct. Specifically, it was closely associated with questions asking if the patient was terminally ill (*r* = 0.292, *P* < 0.001), whether the patient's cancer is curable (*r* = 0.213, *P* = 0.002), and whether the patient expects to live months or years (*r* = 0.195, *P* = 0.008).

#### Communicator of scan results

During the postvisit interviews, patients were asked: “When you met with your oncology provider, did you discuss the results of your recent scan?” Patients who answered “no” were excluded (*n* = 5). This question was followed by: “If yes, with whom did you discuss the results of your scan?” The response options were as follows: (1) oncologist, (2) oncology fellow, (3) physician's assistant, (4) nurse practitioner, (5) nurse, (6) other, specify, and (7) oncology resident. Patients could select multiple response options. The presence of an oncologist was coded “1” for answers that included response option 1 and “0” for answers that included any response option(s) other than response option 1.

#### Potential confounding influences

Previsit LSIU, the presence of multiple providers during scan results discussions, and scan results were examined as potential confounding influences. Previsit LSIU was assessed using the measure outlined above for LSIU. The presence of multiple providers was evaluated based on whether participants selected multiple communicators of scan results when answering the question described above. Scan results were determined by whether the oncology provider who met with the patient to discuss his or her scan results reported to the interviewer that the patient's disease was progressing, stable, or improving based on the patient's most recent scan (*n* = 106, 55.5%). In the cases where this information was unavailable, scan results were determined based on the patient's report at postvisit of whether his or her oncology provider indicated whether his or her disease was progressing, stable, or improving based on his or her most recent scan (*n* = 85, 44.5%). There was high reliability between what the providers and patients reported the scan results to be (*κ *= 0.719, *n* = 89, *P* < 0.001).

### Statistical analysis

Propensity weighting is a common method for matching groups to promote causal inference for between‐group effects 12 and has been used in similar published studies 5, 6. Propensity weights were applied to reduce the potential influence of between‐group differences in patient sociodemographic characteristics on associations between patients who reported a scan visit discussion with an oncologist present versus absent and postvisit LSIU. Propensity weights matched the “oncologist present” and “oncologist absent” samples on patient age, education, gender, race, ethnicity, health insurance status, marital status, primary cancer, and clinic site to reduce the influence of these potential confounders. Chi‐square tests and *t*‐tests using the propensity‐weighted sample were used to evaluate residual between‐group differences in patient sociodemographic characteristics, and none of these characteristics remained confounders.

To determine whether nonsociodemographic characteristics (i.e., previsit LSIU, scan results, and the presence of multiple providers) were potential confounders, we used chi‐square tests to identify whether the variables were significantly (*P* < 0.05) associated with either the predictor (the presence of an oncologist) or the outcome (postvisit LSIU). We found that previsit LSIU was associated with the presence of an oncologist (*P* = 0.027) and postvisit LSIU (*P* < 0.001). The presence of multiple providers was associated with the presence of an oncologist (*P* < 0.001) but not postvisit LSIU (*P* = 0.907). Scan results were not associated with the presence of an oncologist (*P* = 0.151) or postvisit LSIU (*P* = 0.066). Consequently, previsit LSIU and the presence of multiple providers were included as covariates in the multivariable models, while scan results were not included.

Odds ratios for the associations between the presence of an oncologist and patient postvisit LSIU were estimated using logistic regression models in the propensity‐weighted sample. Multiple logistic regression models were used to estimate the independent effects, expressed as adjusted odds ratios, of the presence of an oncologist while adjusting for previsit LSIU and the presence of multiple providers.

Statistical analysis was conducted using SAS statistical software, version 9.4 (Cary, NC). Statistical inferences were based on two‐sided tests with *P* < 0.05 considered statistically significant.

## Results

Of the 209 patients in the analytic sample, 100 (47.8%) reported they discussed their scan results with an oncologist only, 45 (21.5%) reported they discussed their scan results with an oncologist and a nononcologist provider present, 62 (29.7%) reported they discussed their scan results with a nononcologist only, and 2 (1.0%) reported they discussed their scan results with more than one nononcologist. A total of 41.1% of patients acknowledged that their cancer was late stage during the previsit interview, and 47.8% of patients acknowledged that their cancer was late stage during the postvisit interview. Table 1 shows the sociodemographic characteristics and their associations with the presence of an oncologist with propensity weighting to neutralize statistically significant differences between the oncologist present and absent groups.

**Table 1:** Patient sociodemographic characteristics and their associations with the presence of an oncologist during a scan results discussion in the propensity‐weighted sample

Patient characteristics	Unweighted			*P*	Weighted			*P*
	Overall	Oncologist Absent	Oncologist Present		Overall	Oncologist Absent	Oncologist Present	
	*N*	*n* (%)	*n* (%)		*N*	*n* (%)	*n* (%)	
	209	64 (30.6)	145 (69.4)		207.1	62.6 (30.2)	144.5 (69.8)	
	Mean (SD)	Mean (SD)	Mean (SD)		Mean (SD)	Mean (SD)	Mean (SD)	
Age in years	60.1 (9.7)	58.8 (9.8)	60.7 (9.6)	0.193	60.5 (8.9)	60.7 (8.3)	60.4 (9.1)	0.873
Education in years	14.5 (3.2)	13.6 (3.8)	14.8 (2.8)	0.023	14.4 (3.1)	14.4 (3.7)	14.5 (2.8)	0.816
	***n*** **(%)**	***n*** **(%)**	***n*** **(%)**		***n*** **(%)**	***n*** **(%)**	***n*** **(%)**	
Gender								
Male	68 (32.5)	17 (26.6)	51 (35.2)	0.221	62.0 (30.0)	15.8 (25.3)	46.2 (32.0)	0.339
Female	141 (67.5)	47 (73.4)	94 (64.8)		145.0 (70.0)	46.7 (74.7)	98.3 (68.0)	
Race								
White	177 (84.7)	53 (82.8)	124 (85.5)	0.617	178.4 (86.1)	55.0 (87.9)	123.4 (85.4)	0.634
Non‐White	32 (15.3)	11 (17.2)	21 (14.5)		28.7 (13.9)	7.6 (12.1)	21.1 (14.6)	
Ethnicity								
Latino	19 (9.1)	5 (7.8)	14 (9.7)	0.669	23.5 (11.3)	9.6 (15.3)	13.9 (9.6)	0.239
Non‐Latino	190 (90.9)	59 (92.2)	131 (90.3)		183.6 (88.7)	53.0 (84.7)	130.6 (90.4)	
Insurance status								
Insured	159 (76.1)	40 (62.5)	119 (82.1)	0.002	156.8 (75.7)	47.4 (75.8)	109.4 (75.7)	0.993
Not Insured	50 (23.9)	24 (37.5)	26 (17.9)		50.3 (24.3)	15.2 (24.2)	35.1 (24.3)	
Marital status								
Married	122 (58.4)	33 (51.6)	89 (61.4)	0.185	127.4 (61.5)	39.6 (63.3)	87.8 (60.8)	0.733
Not Married	87 (41.6)	31 (48.4)	56 (38.6)		79.6 (38.5)	23.0 (36.7)	56.7 (39.2)	
Primary cancer								
Lung	65 (31.1)	25 (39.1)	40 (27.6)	0.001	72.8 (35.1)	24.9 (39.8)	47.9 (33.1)	0.416
Gastrointestinal	63 (30.1)	8 (12.5)	55 (37.9)		56.9 (27.5)	13.5 (21.5)	43.4 (30.1)	
Other	81 (38.8)	31 (48.4)	50 (34.5)		77.4 (37.4)	24.2 (38.6)	53.2 (36.8)	
Clinic site								
New England	127 (60.8)	33 (51.6)	94 (64.8)	<0.001	128.9 (62.2)	40.4 (64.6)	88.5 (61.2)	0.802
Mid‐Atlantic/South	28 (13.4)	3 (4.7)	25 (17.2)		26.0 (12.6)	6.4 (10.3)	19.5 (13.5)	
Southwest/West	54 (25.8)	28 (43.8)	26 (17.9)		52.2 (25.2)	15.7 (25.1)	36.5 (25.3)	

SD, standard deviation.

New England: Dana‐Farber/Harvard Cancer Center, Yale Cancer Center.

Mid‐Atlantic/South: Memorial Sloan Kettering Cancer Center, Meyer Cancer Center at Weill Cornell Medicine, Virginia Commonwealth University Massey Cancer Center.

Southwest/West: Parkland Hospital, University of New Mexico Cancer Center, Pomona Valley Hospital Medical Center.

Table 2 displays odds ratios between the presence of an oncologist and patient postvisit LSIU using the propensity‐weighted sample and adjusting for patient previsit LSIU and the presence of multiple providers. These results indicate that patients who discussed their recent scan results with an oncologist present versus an oncologist absent were more likely to understand the late stage of their illness after that discussion (AOR = 2.6; 95% CI = 1.2, 6.0; *P* = 0.021; power = 0.90).

**Table 2:** Adjusted odds ratios between patient postvisit late‐stage illness understanding (LSIU) and the presence of an oncologist during a scan results discussion in the propensity‐weighted sample

Predictors	OR	95% CI	*P*	AOR	95% CI	*P*	*R* ^2^
Oncologist present vs. oncologist absent	2.7	(1.4, 5.0)	0.002	2.6	(1.2, 6.0)	0.021	0.332^a^
Previsit LSIU	–	–	–	16.6	(8.0, 34.4)	<0.001	0.316^b^
Multiple providers	–	–	–	0.7	(0.3, 1.7)	0.449	0.334^c^

Adjusted for previsit LSIU and multiple providers. OR, odds ratio; 95% CI, 95% confidence interval; AOR, adjusted odds ratio.

a Step 2 in stepwise model.

b Step 1 in stepwise model.

c Step 3 in stepwise model.

## Discussion

Results of this study demonstrate that advanced cancer patients were over two‐and‐a‐half times as likely to describe their cancer as late stage if an oncologist was present for their scan results discussion in clinic compared to patients whose scan results were presented by a nononcologist member of the care team. This finding remained after propensity weighting corrected for sociodemographic differences between the patients who met with an oncologist to discuss scan results versus those who did not (i.e., education, health insurance status, primary cancer, and clinic site) and after adjusting for the confounding influences of previsit illness understanding and the presence of multiple providers during scan results discussions. These results suggest that it matters whom on the care team meets with patients in clinic to discuss scan results with advanced cancer patients and that oncologist engagement in these conversations is associated with more accurate patient illness understanding.

Prior research has shown the influence of the content of EoL conversations on patient illness understanding 7, 8 and care at the EoL 6, 13, 14. However, research to date has paid little, if any, attention to whether it matters with whom patients discuss such issues. Our data suggest that advanced cancer patients who discuss scan results with an oncologist are more likely to recognize the late‐stage of their illness. This is critical because illness understanding is associated with EoL care preferences 3, care received 5, and care outcomes 15. Simply put, the better patients understand the reality of their condition (i.e., that they are approaching the end of their life), the better prepared they will be to make informed EoL care choices that are consistent with their values.

There are several potential reasons why patients who discussed scan results with an oncologist were more likely to understand the late stage of their illness. First, oncologists would appear to hold the position of highest authority on the cancer care team, and therefore, their words may have the greatest impact. This is consistent with prior research on the powerful influence of authority on persuasion 9, 10, 11. Future research should investigate the extent to which authority, per se, of the clinician communicator is a factor in promoting illness understanding among advanced cancer patients. Second, patients may have developed greater long‐term trust with their oncologists relative to other members of the care team, possibly due to meeting with the same nononcologist less frequently than meeting with the same oncologist. Additional research should examine the influence of trust on improving illness understanding among patients with advanced cancer.

Differences in experience across care team members may also explain our findings. Research suggests that both oncology fellows and nurses (i.e., those who formed the majority of the “nononcologist” group) may lack sufficient training on how to communicate with patients and their families about EoL issues 16, 17. Oncologists have had more years of experience than oncology fellows and residents, and to the extent that our sample is representative, we found that oncologists engaged in scan results discussions more frequently than did nononcologist providers. Thus, oncologists may have had more practice engaging in disease status discussions with advanced cancer patients than the other care team members, which may have improved their communication skills during scan discussions. Additional research is needed to determine whether differences in experience explain our findings and the extent to which increased training can improve communication with patients who are near death. If future research confirms this hypothesis, nononcologist providers may benefit from additional training in communication of disease status. Such training could be incorporated into training programs or provided in CME courses for current practitioners.

Another potential explanation for our results is that patients may expect to hear the “bad news” (i.e., that these scan results mean the patient's cancer is late stage) from their oncologist. Thus, patients may treat scan results discussions with other members of the care team as the first part of the discussion about their disease progression and be unlikely to draw definitive conclusions about the stage of their cancer before speaking with their oncologist. Additionally, certain clinics may have rules about the extent to which nononcologists should discuss disease progression with patients, limiting the amount of information provided by nononcologists. Furthermore, oncologists may feel greater responsibility for shepherding the care of their patients than other members of the care team and so may more often explain the meaning or clinical implications of scan results (i.e., the cancer is late stage) in addition to describing the results of the scan.

It is notable that oncologists were absent from over 30% of scan results discussions, which highlights both how common this practice is and the need for attention to the consequences that may follow from it. It should be noted that comprehensive cancer centers would be expected to employ more oncologists than noncomprehensive cancer centers, and because comprehensive cancer centers were overrepresented in our sampling of patients, we believe the prevalence of nononcologist scan discussions to be a conservative estimate. At the Southwest/West clinics, oncologists were absent from about 52% of scan results discussions, which was a higher rate than at the Mid‐Atlantic/South and New England clinics. A possible explanation for this is that the Southwest/West region had more sites that were not comprehensive cancer centers, and oncologists may have been less readily available to meet with patients at these medical centers than at the other clinics. Future research should attempt to examine how cancer center type (i.e., comprehensive cancer center vs. not) influences scan results discussions. However, it is important to note that propensity weighting corrected the clinical differences in our analyses and our results held in the propensity‐weighted sample.

Our study has several strengths, one of which is its unique design. It is a longitudinal study where advanced cancer patients were interviewed before and after scan results discussions with oncology providers. This allowed us to examine communication factors associated with changes in patients' understanding of their disease. The study also capitalizes on a prospective design in which selection biases that often confound retrospective studies 18 and clinical trials 19 are minimized. Another strength is that these data are not hypothetical and represent the prognostic understanding of advanced cancer patients who are actually confronting death.

Our study also had limitations that should be considered when interpreting the results. First, the “cancer stage” question used in our analyses represents only one element of patients' illness understanding. Still, patients' acknowledgment of late‐stage disease was highly correlated with other illness understanding indicators and was a key outcome in our recent report examining the impact of prognostic disclosure on prognostic understanding 7. Second, oncologists recruited patients to participate whom they believed met the eligibility criteria, and as a result, we do not know the exact total of potentially eligible patients. Future research should confirm these findings in a sample that did not involve oncologists in the recruitment of patients and where the total number of potentially eligible patients is documented. Third, our results should be confirmed in a sample that is less educated and more non‐white and Latino, as the analytic sample was more likely to be more highly educated, white, and non‐Latino. Additionally, data on with whom the patients discussed scan results with were patient reported and not provider reported. Although provider reports may be more accurate, patient reports may better reflect what patients actually heard, which may be of greater relevance to patient prognostic understanding. Finally, we do not have data on the time since patients were diagnosed with advanced cancer. However, all patients had metastatic cancer that had progressed on at least one prior line of chemotherapy and had previous scan results demonstrating they had advanced cancer before being enrolled in the study.

Additional research is needed to investigate what was said during the scan results discussions with providers. Examining the language used by providers will enable us to identify effective communication strategies, which could be incorporated into clinical training programs. Future research should also compare the impact of oncologists versus palliative care physicians on patient's illness understanding following EoL discussions. Like oncologists, palliative care physicians are senior, authoritative physicians who address illness understanding and EoL care issues with advanced cancer patients.

In conclusion, this study found that advanced cancer patients better acknowledge that they have late‐stage disease when an oncologist is present for a scan results discussion in clinic. Our findings suggest it is important for oncologists to engage in scan results discussions directly with advanced cancer patients. Oncologists who are present in clinic to discuss scan results have patients who better understand the late stage of their disease and who are thus better equipped to make informed EoL care choices.

## Conflict of Interest

None declared.

## Supporting information


**Table S1.** Characteristics of patients included in and excluded from the analytic sample.
