# Supplementary material for: Being present: oncologists' role in promoting advanced cancer patients' illness understanding
Source: Cancer Med. 2018 Feb 26;7(4):1511–8. doi: 10.1002/cam4.1389 (PMC5911627; doi:10.1002/cam4.1389)
Supplement: Supplementary file 1 — Table S1. Characteristics of patients included in and excluded from the analytic sample. [file CAM4-7-1511-s001.docx]

| **Supplemental Table. Characteristics of patients included in and excluded from the analytic sample** | | | | | | | |
| --- | --- | --- | --- | --- | --- | --- | --- |
|  |  | **Overall** |  | **Analytic Sample** | | **Excluded** |  |
|  |  | **N** |  | | **n (%)** | **n (%)** |  |
|  |  | 386 |  | | 209 (54.1) | 177 (45.9) |  |
|  |  |  |  |  | |  |  |
| **Patient Characteristics** |  | **mean (SD)** |  | **mean (SD)** | | **mean (SD)** | **P** |
|  |  |  |  |  | |  |  |
| Age in years |  | 60.5 (10.6) |  | 60.1 (9.7) | | 60.7 (11.8) | 0.578 |
| Education in years |  | 13.3 (4.0) |  | 14.5 (3.2) | | 13.5 (3.9) | 0.013 |
|  |  |  |  |  | |  |  |
| **Patient Characteristics** |  | **n (%)** |  | **n (%)** | | **n (%)** | **P** |
|  |  |  |  |  | |  |  |
| Gender |  |  |  |  | |  | 0.284 |
| Male |  | 133 (34.9) |  | 68 (32.5) | | 65 (37.8) |  |
| Female |  | 248 (65.1) |  | 141 (67.5) | | 107 (62.2) |  |
| Race |  |  |  |  | |  | 0.005 |
| White |  | 294 (79.5) |  | 177 (84.7) | | 117 (72.7) |  |
| Non-White |  | 76 (20.5) |  | 32 (15.3) | | 44 (27.3) |  |
| Ethnicity |  |  |  |  | |  | <.001 |
| Latino |  | 57 (14.8) |  | 19 (9.1) | | 38 (21.7) |  |
| Non-Latino |  | 327 (85.2) |  | 190 (90.9) | | 137 (78.3) |  |
| Insurance Status |  |  |  |  | |  | 0.242 |
| Insured |  | 280 (73.7) |  | 159 (76.1) | | 121 (70.8) |  |
| Not Insured |  | 100 (26.3) |  | 50 (23.9) | | 50 (29.2) |  |
| Marital Status |  |  |  |  | |  | 0.601 |
| Married |  | 215 (57.2) |  | 122 (58.4) | | 93 (55.7) |  |
| Not Married |  | 161 (42.8) |  | 87 (41.6) | | 74 (44.3) |  |
| Primary Cancer |  |  |  |  | |  | 0.363 |
| Lung |  | 116 (30.9) |  | 65 (31.1) | | 51 (30.5) |  |
| Gastrointestinal |  | 124 (33.0) |  | 63 (30.1) | | 61 (36.5) |  |
| Other |  | 136 (36.2) |  | 81 (38.8) | | 55 (32.9) |  |
| Clinic Site |  |  |  |  | |  | <.001 |
| New England |  | 183 (47.4) |  | 127 (60.8) | | 56 (31.6) |  |
| Mid-Atlantic/South |  | 73 (18.9) |  | 28 (13.4) | | 45 (25.4) |  |
| Southwest/West |  | 130 (33.7) |  | 54 (25.8) | | 76 (42.9) |  |
| Oncologist presence during scan discussion |  |  |  |  | |  | 0.148 |
| Oncologist absent |  | 71 (28.9) |  | 64 (30.6) | | 7 (18.9) |  |
| Oncologist present |  | 175 (71.1) |  | 145 (69.4) | | 30 (81.1) |  |
| Pre-visit LSIU |  |  |  |  | |  | 0.978 |
| Inaccurate understanding |  | 214 (58.8) |  | 123 (58.9) | | 91 (58.7) |  |
| Accurate understanding |  | 150 (41.2) |  | 86 (41.1) | | 64 (41.3) |  |
| Post-visit LSIU |  |  |  |  | |  | 0.173 |
| Inaccurate understanding |  | 122 (50.4) |  | 109 (52.2) | | 13 (39.4) |  |
| Accurate understanding |  | 120 (49.6) |  | 100 (47.8) | | 20 (60.6) |  |
| NOTE: Patients were excluded from the analytic sample due to drop out and missing data  Abbreviation: SD, standard deviation; LSIU, late-stage illness understanding  New England: Dana-Farber/Harvard Cancer Center, Yale Cancer Center  Mid-Atlantic/South: Memorial Sloan Kettering Cancer Center, Meyer Cancer Center at Weill Cornell Medicine, Virginia Commonwealth University Massey Cancer Center  Southwest/West: Parkland Hospital, University of New Mexico Cancer Center, Pomona Valley Hospital Medical Center | | | | | | | |
